# Inferred Causal Mechanisms of Persistent FMDV Infection in Cattle from Differential Gene Expression in the Nasopharyngeal Mucosa

**DOI:** 10.3390/pathogens11080822

**Published:** 2022-07-22

**Authors:** James J. Zhu, Carolina Stenfeldt, Elizabeth A. Bishop, Jessica A. Canter, Michael Eschbaumer, Luis L. Rodriguez, Jonathan Arzt

**Affiliations:** 1 Foreign Animal Disease Research Unit, Plum Island Animal Disease Center, Agricultural Research Service, United States Department of Agriculture, Orient, NY 11957, USA; carolina.stenfeldt@usda.gov (C.S.); elizabeth.bishop@usda.gov (E.A.B.); jessica.canter@usda.gov (J.A.C.); luis.rodriguez@usda.gov (L.L.R.); 2 Department of Diagnostic Medicine/Pathobiology, Kansas State University, Manhattan, KS 66506, USA; 3 Plum Island Animal Disease Center Research Participation Program, Oak Ridge Institute for Science and Education (ORISE), Oak Ridge, TN 37830, USA; 4 Institute of Diagnostic Virology, Friedrich-Loeffler-Institut, 17493 Greifswald-Insel Riems, Germany; michael.eschbaumer@fli.de

**Keywords:** foot-and-mouth disease virus, FMDV, persistent infection, microarray analysis, differential gene expression, pharyngeal epithelia, AHR, HIF1A, CD39, mucosal immunity

## Abstract

Foot-and-mouth disease virus (FMDV) can persistently infect pharyngeal epithelia in ruminants but not in pigs. Our previous studies demonstrated that persistent FMDV infection in cattle was associated with under-expression of several chemokines that recruit immune cells. This report focuses on the analysis of differentially expressed genes (DEG) identified during the transitional phase of infection, defined as the period when animals diverge between becoming carriers or terminators. During this phase, Th17-stimulating cytokines (IL6 and IL23A) and Th17-recruiting chemokines (CCL14 and CCL20) were upregulated in animals that were still infected (transitional carriers) compared to those that had recently cleared infection (terminators), whereas chemokines recruiting neutrophils and CD8+ T effector cells (CCL3 and ELR+CXCLs) were downregulated. Upregulated Th17-specific receptor, CCR6, and Th17-associated genes, CD146, MIR155, and ThPOK, suggested increased Th17 cell activity in transitional carriers. However, a complex interplay of the Th17 regulatory axis was indicated by non-significant upregulation of IL17A and downregulation of IL17F, two hallmarks of TH17 activity. Other DEG suggested that transitional carriers had upregulated aryl hydrocarbon receptor (AHR), non-canonical NFκB signaling, and downregulated canonical NFκB signaling. The results described herein provide novel insights into the mechanisms of establishment of FMDV persistence. Additionally, the fact that ruminants, unlike pigs, produce a large amount of AHR ligands suggests a plausible explanation of why FMDV persists in ruminants, but not in pigs.

## 1. Introduction

Foot-and-mouth disease (FMD) is one of the most contagious and economically devastating viral diseases of livestock; the disease is caused by FMD virus (FMDV), a positive-sense single-stranded RNA virus of the family *Picornaviridae* (genus *Aphthovirus*). Susceptible hosts include domestic and wild cloven-hoofed animals such as ruminants and pigs. Infection in cattle begins in the respiratory tract. During this primary infection, the virus replicates locally in the nasopharynx or lungs, depending on the route of exposure [1,2]. The infection subsequently spreads via systemic circulation (viremia) to secondary replication sites causing typical vesicles in the oral cavity, on the feet, and other sites of non-haired skin. Mortality is generally low in adult animals, but persistent infection can occur for long periods (30 days–5 years) in 50–80% of infected ruminants [3,4,5,6,7]. In contrast, persistent infection does not occur in pigs [8].

The site of persistent FMDV infection in cattle has been localized to the epithelial cells of the nasopharyngeal mucosa, including the dorsal soft palate and roof of the nasopharynx [6,9,10,11]. Existing FMD vaccines do not prevent or cure persistent infection of pharyngeal epithelial cells [12]. Interestingly, in one set of experiments, FMDV in the oesophageal–pharyngeal fluid of persistently infected cattle was undetectable after dexamethasone treatment; however, virus levels returned to pretreatment levels after cessation of dexamethasone treatment [13]. Currently, the causal mechanisms of FMDV persistence are unknown.

To understand the mechanisms involved in FMDV persistence, a previous study applied an experimental, hypothesis-free functional genomics and bioinformatics approach to identify candidate mechanisms based on genes differentially expressed in tissues targeted and not targeted for persistent FMDV infection [14] and between the targeted tissues of carriers and non-carriers [15,16,17]. In that previous work, differential gene expression in nasopharyngeal tissues of carriers and non-carriers provided early evidence that type 1 regulatory T cells (Tr1) might play a role in persistent infection [15]. Further transcriptomic investigation using micro-dissected nasopharyngeal epithelia suggested that persistent FMDV infection was associated with compromised apoptosis and a reduced cellular immune response [16]. The continued analysis of the differentially expressed genes (DEG) in micro-dissected epithelia during persistent infection indicated that differential gene expression could affect the recruitment of neutrophils, antigen-experienced T cells and/or dendritic cells (DC), natural killer (NK) cell cytotoxicity, and the Th17 response in persistently FMDV-infected carriers [17]. The lung (a non-targeted tissue) was found to express significantly higher levels of TNF cytokines and their receptors than the pharyngeal tissues [14].

The current study provides further analysis of DEG from previously published data [16] collected during the transitional phase of infection that spans the period from acute to persistent infection. The main objective of this study was to infer potential causative factors and mechanisms of establishing FMDV persistent infection in cattle. Using a systems biology approach, we describe several hypothetical mechanisms for the establishment of persistent FMDV infection based on DEG in nasopharyngeal tissues, including contributory roles for aryl hydrocarbon receptor (AHR) ligands, leukocyte function, signaling pathways, and cytokines, chemokines, and their associated receptors.

## 2. Results

### 2.1. Pathway and Gene Ontology Term Analysis

The probes with differential expression at FDR ≤ 0.1 showed that 1274 and 598 known genes were upregulated and downregulated, respectively, in nasopharyngeal epithelia of transitional carriers compared to terminators. The functional analysis of the upregulated DEG using DAVID tools detected significant enrichment in an immune-related gene ontology (GO) term (GO:0006955) and seven KEGG immune processes related to infection in T cells and epithelia, immune cell migration, phagocytosis, and four KEGG signaling pathways involved in immune regulation including (1) PI3K-Akt, (2) NFκB, (3) HIF-1, and (4) Wnt signaling pathways (Table 1). The downregulated genes did not reveal any significant pattern in the same analyses.

**Figure 1:**
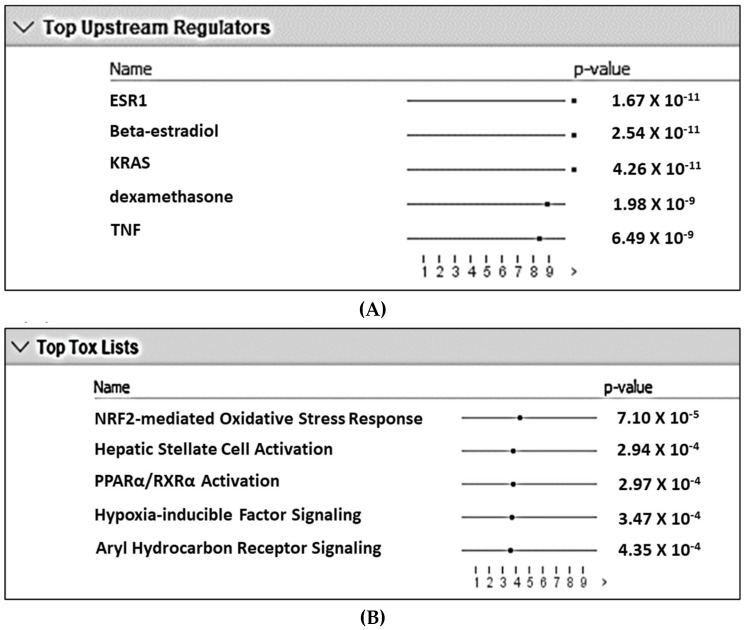
Top five upstream regulators (**A**) and top five biological processes/signaling pathways involved in toxicity (**B**) with the lowest likelihoods (i.e., *p*-value) of the associations/overlaps between the differentially expressed gene set (both up- and downregulated) and the pathways/biological processes by random chances in the Qiagen Ingenuity Pathway Analysis using the list containing ENTREZ numbers and up- and downregulated DEG. The dots in horizontal lines are the negative log transformation of *p*-values.

Analysis of the DEG gene list using the IPA pathways analysis identified the top five inferred upstream regulators for differential expression as (1) estrogen receptor 1 (ESR1), (2) beta-estradiol (an estrogen hormone), (3) KRAS, (4) dexamethasone, and (5) TNF (Figure 1A). Similarly, the top five toxicity-inducing biological processes or signaling pathways were (1) Nrf2-mediated oxidative stress response, (2) hepatic stellate cell activation, (3) PPAR-RXR activation, (4) hypoxia-inducible factor (HIF) signaling, and (5) aryl hydrocarbon receptor (AHR) signaling (Figure 1B). Among the top five upstream regulators, dexamethasone is widely used as an immunosuppressive/anti-inflammatory corticosteroid. Among these top five toxicity-inducing pathways, HIF1α and AHR signaling are mediated by two transcription factors that compete to form heterodimers with ARNT and play a critical role in regulating mucosal immunity [18,19]. NFκB signaling is crucial for the immune response [20]. On this basis, AHR, HIF1A, and NFκB signaling pathways were explored in more detail.

### 2.2. AHR and HIF1α Signaling

AHR and HIF1α compete to form heterodimers with aryl hydrocarbon receptor nuclear translocators (ARNTs). AHR was expressed at a significantly higher level (8.1-fold), and HIF1α expression was significantly downregulated by 1.8-fold in transitional carriers compared to terminators (Table 2). Among three ARNTs, ARNTL expression was the highest and was at a higher, although not significantly increased level (*p* = 0.05) in transitional carriers than terminators. The expression level of eight genes known to be induced by AHR signaling including B7H4 [21], CCL20 [22], CD8A [23,24], CD39 [25,26], CYP1B1 [19], IL6 [22,27], IL23A [28], and STAT3 [28] were significantly higher in transitional carriers than in terminators. A transcript variant of CD39 with a longer 3′ non-coding sequence was also expressed at a higher level very close to significance (FDR = 0.11). Three AHR target genes, CYP1A1, CYP1A2, and IL33 [29], were also expressed at higher gene levels (*p* ≤ 0.05).

HIF1 α expression is inducible by STAT3 and NFκB [30]. Three inhibitors of STATs (PIAS2, PIAS3, and PIAS4) were significantly upregulated at or close to significant levels in transitional carriers (DEG in NFκB signaling are listed in Table 3). PDK1 expression level, inducible by HIF1A [31,32], was 37.6 times lower in transitional carriers than in terminators, whereas three key inhibitors of HIF1α, HIF1AN, LIMD1, and VHL [30,33] were expressed at higher levels (*p* = 0.04, FDR= 0.05 and *p* = 0.03, respectively) in carriers than in terminators. In contrast to enhancing glucose uptake and glycolysis of HIF1A, an enzyme (ACSS3) that catalyzes the first reaction of fatty acid metabolism was upregulated 9.6-fold. HIF1A is activated by the AKT-mTOR signaling pathway via extracellular ATP and TCR signaling [34]. Although AKT1 and AKT2 were upregulated in transitional carriers, two inhibitory genes (PIK3IP1 and TSC1) of this signaling pathway [35,36] were also significantly upregulated. These results indicate reduced HIF1α signaling but increased AHR signaling in the epithelia of transitional carriers, which could impact mucosal immune response.

### 2.3. NFκB Signaling

Several genes playing a critical role in NFκB signaling were differentially expressed in nasopharyngeal tissues between transitional carriers and terminators (Table 3). IKBKB is an indispensable Iκ kinase of the trimeric IκB kinase (IKK) complex in the canonical NFκB pathway [20]. IKBIP is an IKBKB interacting protein. OTUB1 enhances canonical NFκB signaling [37] but inhibits activation of the non-canonical signaling by de-ubiquitination of TRAF3 [38,39]. TGFB2-OT1 increases the LARP1 level to promote the activation of canonical transcription factors [40]. The expression of IKBKB, IKBIP, NOD2, OTUB1, and TGFB2-OT1 was significantly downregulated in transitional carriers compared to terminators. On the other hand, NFKBIA is an inhibitor of the dimerization of transcription factors p50 and RELA in the canonical pathway [20,41]. Additionally, IFRD2 [42], LCOR [43,44], LRRC33 [45], NLK [46], PGRN [47], SIGLEC11 [48], and TRAF1 [49] have suppressive effects on canonical NFκB signaling. The expression of these genes was significantly upregulated in transitional carriers (Table 3).

On the other hand, the transcription factors, receptors, or receptor ligands in non-canonical NFκB signaling such as RELB, NFKB2/p100, CD27, LTB, LTBR, and TNFRSF8/CD30 [20] were expressed at significantly higher levels in transitional carriers. Other genes involved in non-canonical NFκB signaling such as MAP3K14/NIK, CD40LG, RANKL, and TNFRSF1B [20] were also upregulated at *p* ≤ 0.05. Complement membrane attack complexes can activate non-canonical NFκB by forming an Akt+ NIK+ signalosome on Rab5+ endosomes [50]. These results indicate increased non-canonical NFκB signaling and suppressed canonical signaling in the epithelia of transitional carriers. This may promote immune tolerance by inducing tolerogenic DC and Treg cells and suppressing the Th17 response [20,41,51].

### 2.4. Wnt Signaling

The expression of five Wnts (WNT4, WNT5A, WNT7A, WNT10B, and WNT16) was significantly higher in transitional carriers than in terminators (Table 4), and WNT3 was at a higher level (*p* = 0.02). These results, together with the Wnt signaling pathway significantly overrepresented by DEG in the KEGG pathway analysis (Table 1), indicated increased Wnt signaling in carriers. This may indicate induction of tolerogenic DC that can inhibit Th17 and CD8+ cytotoxic T cell activity and promote Treg development, as described previously [52,53,54].

### 2.5. Cytokines and Cytokine Receptors

Expression levels of IL6, IL16, IL23A, IL34, and TNFSF15 were significantly higher (2.5-, 7.2-, 9.1-, 4.4-, and 10.8-fold higher, respectively) in transitional carriers compared to terminators (Table 5). Of these cytokines, IL6 and IL23 promote Th17 differentiation and inhibit the induction of Treg cells in the mucosal immune response [55,56,57]. However, the expression of three cytokines (IL17A, IL17F, and IL22) produced by Th17 cells was not upregulated. IL16 had the highest expression level based on average signal intensities. IL16 recruits CD4-expressing immune cells, preferentially Treg cells [58,59], enhancing the immunosuppressive effect of IL-10 [60], and inducing tolerogenic DC. IL34 is a cytokine of Treg cells [61] and promotes pathogen persistence [62], organ transplant tolerance, immunosuppressive macrophages, and macrophage-M2 polarization [62,63,64,65]. TNFSF15 has diverse functions, including promoting Th2 and Treg response [66,67]. Three Th17 suppressing cytokines (IL21, IL24, and IL33) were also upregulated at the gene levels (*p* < 0.05). These results suggest that the transitional carriers expressed higher levels of both Th17 stimulatory and Th17 suppressive cytokines, which may explain why three Th17 cytokines were not upregulated in carriers.

There were also eight cytokine receptors (ACVR1B, IL18RAP, IL17RB, IL27RA, sIL10RB, TGFBR3, TNFRSF6B, and TNFRSF8) that had significantly upregulated expression and two (ACVR2B and SIGIRR) that were downregulated in transitional carriers (Table 5). Among these receptors, IL27RA was expressed at a very high level. IL27, the ligand of IL27RA, inhibits the differentiation of Th17 cells and IL-17 production [68] and, together with AHR, promotes Tr1 cell differentiation [69,70,71,72]. ACVR1B, ACVR2B, and TGFBR3 are receptors of the TGFβ superfamily, which promote differentiation of Treg, Th17, and/or follicular helper T cells (Tfh) [73,74]. IL17RB, part of the IL25 receptor complex, promotes differentiation of Th2 cells [75]. IL10RB is a co-receptor for IL-10 and IFNλ signaling, but the function of IL10RB without a transmembrane domain (sIL10RB) is unknown. SIGIRR inhibits IL-33-mediated signaling [76]. TNFRSF6B, a soluble decoy receptor, can skew T cell and macrophage differentiation towards Th2 and M2 phenotypes, respectively, and suppress Th17 immune response [77,78]. TNFRSF8 inhibits the proliferation of CD8+ effector T cells [79]. The results of expression of these receptors suggest that the transitional carriers could have suppressive effects on Th17 and CD8+ effector cells and stimulatory effects on Th2 and Treg cells in the epithelia compared to terminators.

### 2.6. Chemokines and Chemokine Receptors

There was significant differential expression of chemokines in epithelia of transitional carriers compared to terminators. Six chemokines, CCL11, CCL14, CCL20, CXCL12, CXCL14, and CXCL13, were significantly upregulated in nasopharyngeal epithelia of transitional carriers, and one (CCL3) was downregulated (Table 6). CCL11, CCL20, CXCL12, and CXCL14 were expressed at >13-fold higher level in transitional carriers than terminators, whereas the remainders were < 3-fold higher. CCL11 selectively recruits eosinophils and mast cells [80]. CCL14 recruits a specific subset of CD4+CD146+CCR5+ Th17 cells [81], whereas CCL20 primarily recruits Th17 cells via the CCR6 receptor [82,83]. CXCL12 polarizes Th to Treg cells and macrophages to M2 activation and recruits antiviral CD8+ T cells [84,85,86]. DPP4, which converts CXCL12 to a CXCL12 antagonist [87], was significantly upregulated in transitional carriers. CXCL13 is a chemoattractant for B cells [88]. CXCL14 was the most differentially expressed chemokine with the highest signal intensity among chemokine DEGs (Table 4). CXCL14 primarily chemoattracts monocytes [89,90]. It also recruits immature DC, M2 macrophages, neutrophils, NK cells and B cells [90,91], and Treg cells [92] and has anti-inflammatory and anti-CXCL12 activities [93,94,95]. CCL3 attracts neutrophils, macrophages, naive CD8+ T cells, and NK cells through binding to the receptors CCR1 and CCR5 [96,97,98]. The total expression of neutrophil-recruiting ELR+ CXCLs, including CXCL1, 2, 3, 5, 8, and 15, was 3.2-fold lower in transitional carriers than in terminators (Table 6).

Three chemokine receptors, CCR1, CCR6, and CCR7, were upregulated in transitional carriers compared to terminators (Table 6). CCR1, CCR3, and CCR5 are expressed on monocyte and macrophages [97]. Upregulated CCR1, together with increased CCR3 and CCR5 (*p* = 0.01 and 0.06, respectively), supports the increased recruitment of monocytes. Upregulated CCR6, the receptor of CCL20, strongly suggests increased Th17 recruitment in transitional carriers. Similarly, CCR3 (high-affinity receptor of CCL11) [99] and CXCR4 (the receptors of CXCL12 and CXCL14) [94,95,100] also showed upregulated expression (*p* ≤ 0.05) in transitional carriers. CCR7 is expressed on naive T and B cells, central memory T cells (Tcm), and mature DC [97]. The expression of two receptors, ACKR3 and XCR1, was significantly downregulated in transitional carriers (Table 4). ACKR3 binds and degrades CXCL12 [101], while XCR1 enhances CD8+ DCs in activating CD8+ T cell-mediated defense via antigen cross-presentation [102]. The receptors of ELR+ CXCLs, CXCR1/CXCR2, were also downregulated in transitional carriers (*p* = 0.03). Therefore, the results of chemokines and their receptors suggest that the epithelia of transitional carriers recruited more monocytes, eosinophils, and Th17 cells, reduced recruitment of neutrophils and CD8+ T cells, decreased antigen-cross presentation to CD8+T cells, and promoted Th17 to Treg transition and macrophage M2 activation compared to terminators.

### 2.7. T-Cell-Associated Factors

It appears that CD4+ T cells especially CD4+ CD8αα+ T cells, but not CD8αβ+ T cells, were specifically increased in transitional carriers based on higher expression levels of CD4 (*p* = 0.02), CD8A [103], CD40L (*p* = 0.01) [104], and ThPOK (CD4 T cell-specific transcription factor) and lower levels of CD8B (*p* = 0.08) (Table 7). A marker gene of a specific subset of Th17 cells (CD146) [105] and a microRNA gene highly expressed in Th17 (BIC/mir155) [106,107,108,109] were expressed at significantly higher levels in transitional carriers than in terminators, whereas the Th17-specific transcription factor RORC was expressed at a higher gene level (*p* = 0.03). However, there are three Th17-suppressing DEG (LXRA; STAT5A and TNFRSF6B) [110,111,112] whose expression was significantly upregulated in transitional carriers compared to terminators. Another Th17-suppressing gene (CD69) [111] was also expressed at a higher gene level (*p* = 0.01) in transitional carriers (Table 7).

Treg marker genes such as FOXP3 (Table 7) and IL10 (Table 5) were not differentially expressed between transitional carriers and terminators; however, several marker genes of type 1 regulatory (Tr1) T cells (GITR, IRF4, IL21, LAG3, and TNFRSF9) were expressed at higher gene levels (*p* ≤ 0.05) in transitional carriers (Table 5 and Table 7). Four upregulated DEG mediating Treg immunosuppressive activities (ADCY4, BTLA, CD39, and GIMAP5) were expressed at significantly higher levels in transitional carriers (Table 2 and Table 7). ADCY4 catalyzes the production of cAMP, an immune suppressive mediator of Treg cells [113,114]. BTLA is a marker of exhausted T cells [115] and promotes peripheral Treg cell differentiation and immune tolerance [116]. GIMAP5 plays a central role in maintaining peripheral tolerance and T cell homeostasis in the gut [117,118,119]. These results suggest that Th17 cell activity may be suppressed in transitional carriers despite increased cell recruitment to the epithelial region.

### 2.8. Myeloid Cell-Associated Factors

The expression of eight genes with immune inhibitory effects on macrophages or antigen-presenting cells (APC) (CD83, CD300D, EMR1, MFSD6, SIGLEC11, SIGLEC15, TIMD4, and TLR2) was significantly upregulated in transitional carriers compared to terminators (Table 8). Signaling through cell-membrane-associated CD83 appears to suppress functions in various immune cell populations [120], and soluble CD83 inhibits human monocyte differentiation into dendritic cells [121]. CD300 proteins are macrophage-specific receptors with regulatory effects [122,123]. CLEC1A dampens dendritic cell activation and downstream Th17 responses [124]. EMR1 mediates the induction of antigen-specific efferent regulatory T cells in peripheral tolerance [125]. MFSD6 is a mediator of MHC haplotype-dependent but not MHC-unrestricted cytotoxicity of macrophages [126]. SIGLEC11 and SIGLEC15 are mainly expressed on macrophages and have an immunosuppressive effect on macrophages [48,127]. TIMD4, expressed only on APC including macrophages, mediates the removal of antigen-specific T cells during the contraction phase of the adaptive immune response [128,129]. TLR2 is a Toll-like receptor that can also induce immune tolerance [130,131,132,133,134]. On the other hand, MFSD6 recognizes certain MHC-I molecules and mediates MHC-I restricted killing by macrophages [126]. MFSD6 expression was 4.1 times lower in transitional carriers compared to terminators (Table 6). These results suggest increased activity of immunosuppressive macrophages and/or dendritic cells in transitional carriers.

### 2.9. Innate Immunity

Transitional carriers had a generally downregulated expression of defensin genes with two genes (DEFB1 and DEFB103A) significantly downregulated by 3.4- and 10.7-fold and one defensin gene (DEFB4B) significantly upregulated by 10.8-fold in transitional carriers (Table 8). DEFB103A is a broad-spectrum antimicrobial and has anti-picornavirus activity [135,136], which played a role in FMDV persistence. NID1 (a soluble NCR2 ligand with NK cell suppressing activity) [137] and MADD (an apoptosis-inhibiting gene) [138] were expressed at significantly higher levels (12.1-and 9.7-fold higher, respectively) in transitional carriers than those in terminators (Table 9).

## 3. Discussion

Historically, it has been reported that approximately 50% of FMDV-infected ruminants remain persistently infected 28 days after infection [3,4,5]; however, experimental studies have shown that the proportion of carriers is often substantially higher [6,11].

Persistent infection does not occur in pigs [8], indicating the involvement of host-specific factors in determining the divergence between FMDV carriers and terminators. The immune mechanisms inferred in this study are consistent with several hypothesized mechanisms identified in nasopharyngeal tissues during persistent infection [17], including (1) reduced recruitment of neutrophils and CD8+ T effector cells, (2) suppressed NK and macrophage cytotoxicity via downregulated MFSD6 and NID1, and (3) suppression of the Th17 response and canonical NFκB signaling pathway. Additionally, previous work demonstrated that expression of chemokines that recruit neutrophils and CD8+ T effector cells was reported to be significantly lower in pharyngeal tissue than in the lung, where primary, but not persistent, infection occurs [14,139], indirectly supporting the involvement of these chemokines in preventing FMDV persistent infection.

The differential expression of ELR+ CXCLs and CCL3 is consistent with the microscopic analyses of the nasopharynx of the animals included in this study, wherein there were reduced quantities of CD8+ T cells in the epithelia of transitional carriers compared to terminators [16]. These results suggested the importance of neutrophil and CD8+ T effector cell recruiting chemokines in FMDV clearance, given that CD8+ cytotoxic T cells kill infected cells and neutrophils can clear virus infection via phagocytosis and extracellular traps [140]. This also agrees in part with the finding from one study that dexamethasone injection inhibited FMDV production in the oesophageal–pharyngeal fluid of persistently infected cattle but did not cure the infection [13]. This is based on dexamethasone treatment causing neutrophilia, lymphopenia, and eosinopenia in ruminants [141,142,143].

IL-17RA signaling in the epithelium, activated by two Th17-specific cytokines, IL17A and IL17F, is required for neutrophil recruitment. During the transitional phase, the involvement of Th17 cells in the FMDV infection of pharyngeal epithelia was strongly supported by upregulated expression of Th17-promoting cytokines and chemokines (IL6, IL23A, CCL14, CCL20, and CCR6 listed in Table 5 and Table 6) and Th17-associated genes (CD4, CD146, MIR155, TGFBR3, and ThPOK shown in Table 7) in transitional carriers, suggesting that the Th17 response was needed to clear FMDV. However, the expression of Th17 cytokines (IL17A, IL17F, and IL22) was not upregulated in transitional carriers, indicating that the activity of Th17 cells was suppressed, potentially as a result of upregulated Th17 suppressing genes CD39, CD69, IL16; LXRA, STAT5A, TIMD4, and TNFRSF6B (Table 7). The suppression could also be mediated by several upregulated immune suppressive genes including ADCY4, BTLA, GIMAP5, and IL34 based on publications cited herein.

AHR plays a key role in regulating Th17 differentiation and activity [19]. Among CD4+ T cells, AHR expression is restricted to the Th17 cell subset, including Treg cells [144]. Natural AHR agonists enhance Th17 differentiation [145]. Different AHR ligands, such as TCDD and FICZ, induce different effects on Th17 and Treg [146]. Dietary AHR ligands (indole-3-carbinol and 3,3′-diindolylmethane) can cause trans-differentiation of Th17 cells into T cells with regulatory phenotypes during the resolution of inflammation, reduce IL17 expression [147] and induce immune tolerance [148]. Tryptophan derivatives such as indole-3-lactic acid produced by Lactobacillus inducted CD4+CD8αα double positive intraepithelial lymphocytes (DP IELs), which display regulatory functions associated with immune tolerance [149]. Our results suggest increased activity of regulatory T cells, which was supported by upregulated cytokine (IL34) and effector genes (ADCY4, B7H4, CD8A, and CD39) of regulatory T cells. B7H4, CD8A, and CD39 are known to be AHR target genes. Several type 1 regulatory (Tr1) T cell genes (GITR, IL21, IRF4, LAG3, and TNFRSF9) [112], but not Treg marker genes (IL10 and FOXP3), were upregulated (*p* ≤ 0.05) in transitional carriers (Table 5 and Table 7), supporting AHR-induced Th17 trans-differentiation into regulatory T cells. AHR also induces suppressive macrophages and tolerogenic DC to promote the differentiation of Treg cells [21,150,151,152,153,154,155,156]. Our results (Table 8) indicated the induction of suppressive macrophages and tolerogenic DC in carriers.

Effects of AHR signaling can also be mediated through dimerization with other transcription factors such as estrogen receptors [157,158,159], HIF1A [18,160,161,162,163]; NFκB [164], PPAR [165,166]. Interestingly, estrogen receptor, ESR1, was detected as a top up-stream regulator, and DEG were over-represented in the PPAR signaling pathway in this study. HIF1A and AHR compete to form heterodimers with AHR nuclear translocators (ARNTs) and mutually inhibit each other. Reduced HIF1A signaling inhibits IL17 production in CD4+ T cells and cytotoxicity of CD8+ T cells contributing to T cell exhaustion in chronic infections [167]. AHR:RelA dimerization antagonizes the classical NFκB pathway, whereas AHR:RelB enhances non-canonical pathway signaling [164,168]. The DEG listed in Table 3 indicate higher non-canonical and lower canonical NFκB signaling in transitional carriers compared to terminators, according to the review article by Sun (2017), which could suppress the Th17 response according to IL17 signaling in the canonical NFκB pathway [169]. The non-canonical NFκB pathway plays an important role in promoting immune tolerance by inducing tolerogenic DC and Treg cells [20,41], suppressing the Th17 response [51]. Additionally, AHR signaling enhances Wnt signaling [103], and both Wnt and AHR signaling can induce tolerogenic DC [52,54].

Our results demonstrated upregulated AHR and its several target genes and downregulated HIF1A and its target genes in transitional carriers (listed in Table 2). AHR is a promiscuous xenobiotic receptor and ligand-dependent transcription factor that binds to various chemicals such as plant flavonoids, polyphenolics, and indoles as well as to pollutants such as synthetic polycyclic aromatic hydrocarbons and dioxin-like compounds [170]. Interestingly, some short chain fatty acids (SCFA), e.g., propionate and butyrate, are also AHR ligands [171] and can induce AHR expression [172,173] and increase cell response to AHR ligand stimulation [174]. Some of the AHR ligands are produced in the rumen as part of normal ruminant physiology, suggesting an interesting hypothetical mechanism to explain why ruminants, but not pigs are prone to persistent FMDV infections.

It is well-established that B-cell function is altered during the FMDV carrier state. Specifically, anti-FMDV IgA detection in secretions has been reported to be significantly higher in carriers than in non-carriers [175,176]. This indicates chronic stimulation of B cells and suggests that antibodies alone cannot clear the FMDV carrier state, but rather cell-mediated immunity is required. In the current study, B-cell induction was indicated by upregulation of CD21, CD19, and CD81 (the B cell co-receptor complex) and 16 immunoglobulin probes in transitional carriers (data not shown).

In summary, this work supports previous studies that indicated that the establishment and maintenance of the FMDV carrier state are associated with differential gene expression in the nasopharyngeal tissues, known to be the site of persistent infection. Specifically, pathway analysis of DEG suggested that several immune regulatory mechanisms are associated with FMDV persistence. DEG of cytokines, chemokines, and the receptors suggest an increased differentiation and migration of Th17 cells but reduced recruitment of neutrophils and CD8+ T effector cells to the infected tissues of transitional carriers compared to terminators. However, IL17A and IL17F expression were not increased in carriers, indicating complex regulation of the Th17 response. The Th17 response is known to play a key role in inducing the expression of neutrophil recruiting chemokines, which is regulated, in part, by AHR signaling. Upregulated AHR signaling in carriers was also supported by DEG in NFκB and Wnt signaling pathways. This leads us to speculate that AHR ligands produced in the rumen and their effect on various physiological functions might play a role in establishing FMDV persistent infection, which could also explain why FMDV persists in some ruminants but not in pigs.

## 4. Materials and Methods

### 4.1. Study Design and Gene Expression Data

The microarray data used in this study and the design of the animal experiments have been reported previously [16]. All data utilized in this study were derived from microarray-based gene expression profiles of micro-dissected nasopharyngeal epithelia from FMDV-infected cattle during the transitional phase of infection spanned from 12 to 21 dpi. The previous works defined the transitional phase as the period after acute infection but before the defined carrier phase; animals that remained infected during the transitional phase (transitional carriers) consistently progressed to becoming carriers [6].

The data were produced using a custom bovine gene expression 60-mer oligonucleotide microarray as described by Zhu et al. [14]. Microarrays and reagents were manufactured by Agilent Technologies (San Jose, CA, USA), and the lab procedures were conducted based on the protocols and equipment recommended by the manufacturer. For comparison of the gene expression levels between transitional carriers (animals that were still infected) and terminators (animals that had recently cleared infection) during the transitional phase of infection, microarray expression data from the micro-dissected nasopharyngeal epithelia of three animals from each cohort were compared, as previously reported by Stenfeldt et al. [16].

### 4.2. Statistical Analysis

R scripts implemented with the LIMMA package [177] were used to normalize and analyze the microarray data as previously described [15]. All signal intensities (averaged photons per pixel in the microarray images) used in the statistical analysis were Log2 transformed. Genes differentially expressed between transitional carriers and terminators with a false discovery rate (FDR) of 0.10 or smaller and an expression difference of at least 50% were considered statistically significant genes in this study. This FDR threshold increases the detection power (fewer false negatives/type II errors) with a false positive (type I error) rate of 0.10 in declared DEG, or one expected false positive in ten DEG, compared to FDR at 0.05, which balances type I and type II errors.

### 4.3. Pathway Analysis

The methods of pathway analysis of DEG have been described [17]. All bovine genes included in the microarray design were mapped to human reference genes using computer analysis via NCBI BLAST and/or manual annotation by aligning the microarray probe sequences on bovine genome sequences on the UCSC Genome Browser using BLAT (https://genome.ucsc.edu/index.html (accessed on 1 June 2022)). The list of upregulated and downregulated genes associated with each human Entrez Gene ID was analyzed with Ingenuity Pathway Analysis (IPA) (Qiagen, Germantown, MD, USA) and the NCBI Functional Annotation Bioinformatics Microarray Analysis program (DAVID Bioinformatics Resources version 6.8) to identify the biological pathways significantly over-represented among DEG. The biological functions of DEG were determined based on scientific publications (included as cited references) or on the NCBI Gene database (https://www.ncbi.nlm.nih.gov/gene/ (accessed on 1 June 2022)).

### 4.4. Biological Inferences

The biological inferences have been described [17], which were based on (i) reported biological functions of DEG, (ii) differential gene expression including averaged signal intensity and magnitudes (fold difference) of upregulated or downregulated expression, assuming that (1) genes with a higher signal intensity and larger differential expression have a more substantial biological role in their gene group and (2) upregulated expression enhances gene activities and vice versa. Differential expression of genes with cell-specific expression was also used to infer the differences in the number of the cells. Genes with no significant differential expression (FDR > 0.10) but known to play important roles in the relevant biological pathways/processes associated with other DEG were also used as references or supporting results for DEG-related mechanisms. Probabilities of differential expression at gene levels are listed as *p*-values along with the FDR. Expression levels of genes downregulated or upregulated in transitional carriers compared to terminators are shown as negative and positive values (fold changes), respectively. Immune regulatory mechanisms especially involved in mucosal immunity and its association with ruminant physiology were also taken into consideration in the formulation of the hypothesis.

## Figures and Tables

**Table 1:** Gene ontology (GO) term associated with biological processes (GOTERM_BP) and Kyoto Encyclopedia of Genes and Genomes (KEGG) Pathways significantly (*p* value < 0.05 with Benjamini correction for multiple tests) over-represented in transitional carriers compared to terminators.

Pathway	Function	Term	*p* Value
GOTERM_BP	Immunity	GO:0006955~immune response	0.007
	Gene expression	GO:0000184~mRNA nonsense-mediated decay	0.039
		GO:0006364~rRNA processing	0.042
		GO:0006412~translation	0.044
		GO:0006413~translational initiation	0.045
KEGG	Immunity	hsa05166: Human T-cell leukemia virus-I infection hsa04062: Chemokine signaling pathway hsa05100: Bacterial invasion of epithelial cells hsa04670: Leukocyte transendothelial migration hsa04666: Fc gamma R-mediated phagocytosis hsa04662: B cell receptor signaling pathway hsa05169: Epstein-Barr virus infection	1.96 × 10^−4^ 0.002 0.004 0.016 0.021 0.025 0.042
	Gene expression	hsa04151: PI3K-Akt signaling pathway hsa04064: NF-kappa B signaling pathway hsa04066: HIF-1 signaling pathwayhsa04310: Wnt signaling pathway	0.030 0.043 0.044 0.047

**Table 2:** Average expression signal intensity (ESI), false discovery rates (FDR), and fold differences in AHR and HIF1A signaling-related genes that were differentially expressed between the nasopharynx epithelia of transitional carriers and terminators.

Group	Gene	ESI	*p*	FDR	Fold	Function
Transcription factors	AHR	296		0.04	8.1	Activated by AHR ligands
	ARNT	56	0.99	1.00	1.0	Dimerize with AHR and HIF1A
	ARNTL	495	0.05	0.30	6.5	
	ARNTL2	61	0.72	0.93	1.1	
	HIF1A	4537		0.05	−1.8	Activated by eATP, hypoxia, TCR
AHR target genes	B7H4	2468		0.04	5.0	Immune inhibitory receptor
	CD8A	1601		0.05	9.8	CD8αα, inhibit TCR signaling
	CD39	327		0.00	14.5	Adenosine-mediated immune suppression
	CD39_l ^1^	1029		0.11	4.5	
	CYP1A1	176		0.24	3.1	Metabolism of AHR ligands
	CYP1A2	4255		0.31	3.4	
	CYP1B1	48		0.03	2.4	
	CCL20	2161		0.03	18.7	Recruit Th17 to epithelia
	IL6	74		0.01	2.5	Th→Th17 differentiation
	IL23A	1393		0.07	9.1	
	IL33	578	0.04	0.27	4.3	↑ ^2^ Treg differentiation and function
	STAT3	3206		0.00	20.3	Th17 and Treg cell differentiation
HIF1A target genes	ACSS3	197	0.00	0.00	9.6	↑ Fatty acid metabolism
	PDK1	153		0.00	−37.6	↓ Pyruvate metabolism via tricarboxylic acid cycle (TCA)
	PDK1_l ^1^	506	0.01	0.15	−2.9	
Genes regulating HIF1A signaling and expression	AKT1	2544		0.06	2.9	AKT signaling
	AKT2	1402		0.05	2.3	
	HIF1AN	1901	0.04	0.26	1.9	HIF1A inhibitor
	LIMD1	29		0.05	1.7	↑ HIF1A degradation
	VHL	3998	0.03	0.22	2.1	
	PIK3IP1	183		0.08	15.1	↓ AKT-mTOR signaling
	TSC1	3501		0.04	2.1	
	PIAS2	540		0.01	10.4	Inhibitor of STATs
	PIAS3	1536	0.01	0.12	3.8	
	PIAS4	4885	0.01	0.13	2.0	

^l^ denotes longer 3′ end non-coding transcription variant. ^2^ ↓ and ↑ denote inhibiting or inducing, respectively.

**Table 3:** Mean expression signal intensities (ESI) and expression differences (fold) in genes of canonical and non-canonical NFκB signaling pathways between of transitional FMDV carriers and terminators.

Group	Gene	ESI	*p*	FDR	Fold	Function
Canonical pathway	IKBKB/IKKβ	318		0.03	−3.2	Predominant IKK catalytic unit
	IKBIP	1973		0.07	−7.5	IKKβ interacting protein
	NOD2	504		0.08	−6.1	Bind muramyl dipeptide
	OTUB1	2939		0.07	−3.3	Stimulator via stabilizing c-IAP
	TGFB2_OT1	266		0.00	−8.6	Activate NFKB RELA
	IFRD2	14,949		0.03	2.2	Deacetylation of RELA
	IL1R2	1265	0.01	0.17	4.8	Decoy receptor of IL1R1
	LCOR	896		0.00	36.3	Act with PPARG to ↓ NFkB signaling Inhibit TLR signaling
	LRRC33	89		0.00	8.1	
	MAP3K2	2846		0.05	−7.9	MAPK signaling Inhibitor of NFKB1 and RELA Disrupt TAK1 and IKKβ interaction
	NFKBIA	25,742		0.01	2.0	
	NLK	375		0.01	17.5	
	PGRN	4870		0.02	3.8	Inhibit TNF signaling, ↑ Treg
	SIGLEC11	191		0.00	16.6	Suppress LPS signaling
	TRAF1	373		0.00	11.2	Inhibit TLR signaling
Non-canonical pathway	TNFRSF1B	8298	0.01	0.17	3.8	TNF receptor 2
	MAP3K14/NIK	2846	0.05	0.32	4.3	Kinase of non-canonical pathway
	NFKB2/p100	2376		0.03	4.5	Transcription factors of non-canonical pathway
	RELB	11,156		0.05	4.2	
	TNFSF8/CD30L	350	0.07	0.35	2.9	Receptors and ligands of non-canonical pathway
	TNFRSF8/CD30	40		0.09	2.0	
	LTB	10,635		0.08	9.3	
	LTBR	4407		0.03	14.4	
	CD40LG	251	0.01	0.12	11.3	
	CD40	1488		0.05	8.6	
	RANKL/TNFSF11	163	0.02	0.21	3.8	
	CD27/TNFRSF7	556		0.01	9.9	
	OTUB1	2939		0.07	−3.3	Inhibitor via stabilizing TRAF3

**Table 4:** Average expression signal intensities (ESI) and expression differences (fold) in WNT genes between transitional carriers and terminators.

Gene	ESI	*p*	FDR	Fold
WNT4	225		0.05	5.7
WNT5A	285		0.09	4.2
WNT7A	107		0.01	4.2
WNT10B	39		0.04	1.7
WNT16	53		0.00	6.5
WNT3	81	0.02	0.21	2.9

**Table 5:** Average expression signal intensities (ESI), false discovery rates (FDR), and fold differences in cytokines and receptors that were differentially expressed between transitional carriers and terminators.

Group	Gene	ESI	*p*	FDR	Fold	Biological Activity and Expressing Cells ^1^
Cytokines	IL6	74		0.01	2.5	Stimulate Th → Th17 differentiation
	IL16	3000		0.03	7.2	↑ CD4+ cells, ↑ immune tolerance
	IL23A	1393		0.07	9.1	Stimulate Th → Th17 differentiation
	IL34	178		0.02	4.4	Mφ → M2/MDSC and Th → Treg
	TNFSF15	313		0.07	20.8	Activate T cells, Treg expansion
	IL17A	161	0.70	0.92	1.4	Cytokines produced by Th17 cells
	IL17F	227	0.13	0.47	−2.1	
	IL22	16	0.24	0.56	1.1	
	IL10	127	0.95	0.99	1.1	Immune inhibitory cytokine
	IL21	184	0.02	0.17	3.3	Act with IL27 and AHR to ↑ Tr1 IL-10 family, delimit Th17 response
	IL24	2804	0.02	0.17	6.7	
	IL33	578	0.04	0.27	4.3	Inhibit Th17 activity
	IL36A	212	0.01	0.15	3.7	Synergize IL17A
	TGFB1	223	0.26	0.64	1.7	
	TGFB2	151	0.16	0.52	2.2	Th17, Treg, and Tr1 differentiation
	TGFB3	130	0.07	0.35	2.0	
	(MMP9)	871		0.00	37.5	Activate TGFβ to ↑ tolerogenic DC/MDSC
Cytokine Receptors	ACVR1B	793		0.05	10.2	Stimulate Th2 and Treg differentiation
	ACVR2B	108		0.05	−3.9	
	IL17RB	121		0.00	15.6	Stimulate Th2 differentiation
	IL18RAP	118		0.06	2.9	IL-18 signaling
	IL27RA	47,894		0.02	1.9	↓ Th2, Th17, Treg; ↑ Th1, Tr1, ↑ CD39
	sIL10RB	2425		0.06	12.0	IL10 and IFNλ signaling
	SIGIRR	1700		0.02	−9.2	Inhibit signaling of IL-1 cytokines
	TGFBR3	87		0.01	2.5	Th17, Treg, and Tr1 differentiation
	TNFRSF6B	907		0.06	5.2	Suppress IL17 production and FAS
	TNFRSF8	40		0.09	2.0	Inhibit CD8+ effector cells

^1^ MDSC—monocyte-derived suppressive cells; Mo—monocyte; Mφ—macrophages.

**Table 6:** Average expression signal intensities (ESI), false discovery rates (FDR), and fold differences in chemokine and the receptor genes differentially expressed between transitional carriers and terminators.

Group	Gene	ESI	*p*	FDR	Fold	Biological Activity and Expressing Cells ^1^
Chemokines	CCL3	907		0.05	−4.5	Recruit Mφ, NK, CD8+ T cells, neutrophils
	CCL11	1074		0.03	26.7	Recruit eosinophils, mast cells, Th2
	CCL14	54		0.01	2.4	Recruit CD4+CD146+CCR5+ Th17 cells
	CCL19	829	0.01	0.12	2.9	Recruit DC, T, and B cells via CCR7
	CCL20	2161		0.03	18.7	Recruit Th17, B cells, and DC to epithelia
	CCL28	436	0.02	0.19	4.5	IgA-expressing cells
	CXCL12	487		0.00	13.9	Recruit CD8+ T cells; Th1 → Tr1, Mφ → M2
	(DPP4)	1283		0.01	10.6	Convert CXCL12 to antagonist Recruit B cells and Tfh cells
	CXCL13	77		0.06	2.6	
	CXCL14	2727		0.04	34.3	Recruit myeloid and B cells, promote Treg
	CXCL15	525	0.01	0.14	−6.3	Recruit neutrophils
	ELR+CXCLs ^2^	2530		n/a	−3.2	Recruit neutrophil > Mo, NK, CD8+ T cells
Chemokine Receptors	CCR1	1159		0.03	4.5	Mo, Mφ, neutrophil, Th1, DC
	CCR2	171	0.01	0.11	4.4	Mo, Mφ, Th1, iDC, basophil, NK
	CCR3	76	0.02	0.19	8.1	Eosinophil > basophil, mast cell
	CCR5	153		0.34	2.2	Mφ, Th1, NK, Treg, CD8+ T, DC, neutrophil
	CCR6	1976	0.06	0.03	6.9	Th17 > iDC, γδ T, NKT, NK, Treg, Tfh cells naive T and B, mDC, Tcm cells
	CCR7	1213		0.01	16.5	
	CXCR1/2	311		0.24	−1.5	neutrophil > Mo, NK, CD8+ T, mast cell
	CXCR4	7857	0.03	0.31	2.4	CXCL12 and CXCL14 receptor
	ACKR3	685		0.10	−4.4	Bind and degrade CXCL12
	XCR1	629	0.05	0.07	−4.0	CD8+ dendritic cell cross-presentation

^1^ iDC—immature dendritic cells; Mo—monocyte; Mφ—macrophages; IEL—intraepithelial lymphocytes; NKT—natural killer T cells; Trm—resident memory T cells. ^2^ ELR+CXCLs include CXCL1, CXCL2, CXCL3, CXCL5, CXCL8, and CXCL15, in which total signal intensity is the sum of the signal intensity of each ELR+CXCL chemokine in carriers and terminators.

**Table 7:** Average expression signal intensities (ESI), false discovery rates (FDR), and fold differences in T cell-associated genes differentially expressed between transitional carriers and terminators.

Group	Gene	ESI	*p*	FDR	Fold	Biological Activity and Expressing Cells ^1^
CD4+ or CD8+ cells	CD4	58	0.02	0.19	3.3	T helper cell marker
	CD5	6619		0.09	3.0	BTLA → ↑ CD5 to ↑ Treg differentiation
	CD8A	1601		0.05	9.8	Form CD8αα dimer, high on CD4+ IEL
	CD8B	120	0.08	0.39	−2.8	CD8αβ, CD8+ cytotoxic T cells
	CD40L	251	0.01	0.12	11.3	Primarily on activated CD4+ T cells
	TGFBR3	87		0.01	2.5	Th17, Treg, and Tr1 differentiation, Zhang
	ThPOK	3191		0.02	4.0	CD4+ T cell transcription factor
Th17 cells	CD146	118		0.01	2.6	Expressed on a Th17 subset Highly expressed in Th17 and Treg cells
	MIR155	219		0.00	8.1	
	RORC	723	0.03	0.23	6.3	TH17 transcription factor Inhibit Th17 differentiation Inhibit Th17 differentiation
	CD69	268	0.01	0.13	2.3	
	LXRA	1717		0.03	10.1	
	STAT5A	11,302		0.03	1.8	Inhibit Th17 differentiation
	TNFRSF6B	907		0.06	5.2	Suppress IL17 production
Regulatory T cells	CD49B	216	0.14	0.49	1.9	Tr1 marker
	FOXP3	218	0.34	0.73	−1.1	FOXP3+ Treg transcription factor
	GITR	88	0.05	0.31	1.6	TNFRSF18 on Treg cells ↑ Tr1 but ↓ Th17 differentiation
	IRF4	12,334	0.01	0.11	2.5	
	LAG3	214	0.02	0.20	2.5	Tr1 marker
	TNFRSF9	176	0.01	0.11	8.8	Non-specific Tr1 marker
	ADCY4	1005		0.01	36.2	Immune suppression by ↑ cAMP to activate PKA
	ADCY6	498	0.01	0.12	4.7	
	BTLA	80		0.06	7.4	Th cell inhibitory receptor
	GIMAP5	205	0.02	0.03	22.4	Immune tolerance, expressed in T cells

^1^ MDSC—monocyte-derived suppressive cells; Mo—monocyte; Mφ—macrophages.

**Table 8:** Average expression signal intensities (ESI), false discovery rates (FDR), and fold differences in dendritic cells (DC)- and macrophage (Mφ)-expressing genes differentially expressed between transitional carriers and terminators.

Gene	ESI	FDR	Fold	Biological Activity and Expressing Cells ^1^
CD83	11,022	0.10	3.5	Suppressive on several immune cells
CD300D	154	0.04	2.7	Macrophage suppressive receptor
CLEC1A	149	0.04	9.0	DC receptor, ↓ Th17 response
EMR1	84	0.06	2.4	Mφ induce antigen specific Treg cells
MFSD6	2626	0.03	−4.1	MHC-I restricted killing by Mφ
SIGLEC11	191	0.00	16.6	Suppress LPS signaling in macrophages
SIGLEC15	190	0.01	2.1	Suppress Ag-specific T cell responses
TIMD4	5266	0.01	8.8	Remove apoptotic and T effector cells
TLR2	100	0.06	11.9	Promote immune tolerance

^1^ DC—dendritic cell; Mφ—macrophages.

**Table 9:** Average expression signal intensities (ESI), false discovery rates (FDR), and fold differences of innate and humoral immunity-related genes differentially expressed between the nasopharynx epithelia of transitional carriers and terminators.

Group	Gene	ESI	FDR	Fold	Functions
Defensin	DEFB1	516	0.07	−3.4	Antimicrobial defensin
	DEFB4B	8363	0.00	10.8	Anti-Gram - bacteria
	DEFB103A	3069	0.00	−10.7	Anti-Gram - & + bacteria
	DEFB13	2339	0.74	−1.80	Beta-defensin 13
	EDB	2559	0.24	−4.8	Enteric beta-defensin
	LAP	30,838	0.27	−2.1	Lingual antimicrobial peptide
	TAP	23,613	0.28	−3.2	Tracheal antimicrobial peptide
NK cell cytotoxicity	KMT2E	443	0.57	1.6	NCR2 ligand
	MADD	397	0.01	9.7	↓ TRAIL-induced apoptosis
	NID1	346	0.04	8.9	Inhibit NK cell cytotoxicity

## Data Availability

The animal experiments of the microarray studies have been published, and the original raw data are available in the NCBI databases (accession number: GSE104058) (http://www.ncbi.nlm.nih.gov/geo/query/acc.cgi?acc = GSE104058 (accessed on 1 June 2022)). The datasets generated for this study are located at: http://www.ncbi.nlm.nih.gov/geo/query/acc.cgi?acc=GSE104058 (accessed on 1 June 2022).
